# Prognostic relevance of molecular subtypes and master regulators in pancreatic ductal adenocarcinoma

**DOI:** 10.1186/s12885-016-2540-6

**Published:** 2016-08-12

**Authors:** Rekin’s Janky, Maria Mercedes Binda, Joke Allemeersch, Anke Van den broeck, Olivier Govaere, Johannes V. Swinnen, Tania Roskams, Stein Aerts, Baki Topal

**Affiliations:** 1 Laboratory of Computational Biology, KU Leuven Center for Human Genetics, Herestraat 49, 3000 Leuven, Belgium; 2 Department of Abdominal Surgical Oncology, University Hospitals Leuven, KU Leuven, Herestraat 49, 3000 Leuven, Belgium; 3 Nucleomics Core, Flanders Institute for Biotechnology (VIB), KU Leuven, Herestraat 49, 3000 Leuven, Belgium; 4 Department of Pathology, University Hospitals Leuven, KU Leuven, Herestraat 49, 3000 Leuven, Belgium; 5 Laboratory of Lipid Metabolism and Cancer, Department of Oncology, LKI-Leuven Cancer Institute, KU Leuven, Herestraat 49, 3000 Leuven, Belgium

**Keywords:** Pancreatic ductal adenocarcinoma, Molecular subtypes, Master regulators, HNF1A/B

## Abstract

**Background:**

Pancreatic cancer is poorly characterized at genetic and non-genetic levels. The current study evaluates in a large cohort of patients the prognostic relevance of molecular subtypes and key transcription factors in pancreatic ductal adenocarcinoma (PDAC).

**Methods:**

We performed gene expression analysis of whole-tumor tissue obtained from 118 surgically resected PDAC and 13 histologically normal pancreatic tissue samples. Cox regression models were used to study the effect on survival of molecular subtypes and 16 clinicopathological prognostic factors. In order to better understand the biology of PDAC we used *iRegulon* to identify transcription factors (TFs) as master regulators of PDAC and its subtypes.

**Results:**

We confirmed the *PDAssign* gene signature as classifier of PDAC in molecular subtypes with prognostic relevance. We found molecular subtypes, but not clinicopathological factors, as independent predictors of survival. Regulatory network analysis predicted that HNF1A/B are among thousand TFs the top enriched master regulators of the genes expressed in the normal pancreatic tissue compared to the PDAC regulatory network. On immunohistochemistry staining of PDAC samples, we observed low expression of HNF1B in well differentiated towards no expression in poorly differentiated PDAC samples. We predicted IRF/STAT, AP-1, and ETS-family members as key transcription factors in gene signatures downstream of mutated KRAS.

**Conclusions:**

PDAC can be classified in molecular subtypes that independently predict survival. HNF1A/B seem to be good candidates as master regulators of pancreatic differentiation, which at the protein level loses its expression in malignant ductal cells of the pancreas, suggesting its putative role as tumor suppressor in pancreatic cancer.

**Trial registration:**

The study was registered at ClinicalTrials.gov under the number NCT01116791 (May 3, 2010).

**Electronic supplementary material:**

The online version of this article (doi:10.1186/s12885-016-2540-6) contains supplementary material, which is available to authorized users.

## Background

Pancreatic ductal adenocarcinoma (PDAC; also called pancreatic cancer) is one of the most aggressive cancers, associated with a poor prognosis [1]. The lack of early diagnostic markers and efficient therapeutic modalities for PDAC results in extremely poor prognosis. For several decades, many efforts have been undertaken to better understand the pathogenesis and biology of PDAC, and to improve patient survival through early diagnosis and various therapeutic strategies. However, no substantial advances have been made to overcome its lethal destiny. Today, adequate surgical resection is the only chance for patients to be cured from PDAC, often in combination with peri- or post-operative chemo(radio)therapy [2, 3]. Unfortunately, only selected patients with localized disease are potential candidates for surgical management with curative intent. Even in the group of surgically treated curable patients, the majority will develop cancer recurrence and die within two years. Most patients with pancreatic cancer are not eligible for surgery as they present in advanced stages with distant organ metastases and/or locoregional extension. Systemic chemotherapy is the standard of care for patients with advanced inoperable PDAC, resulting in a median survival of about 8 months [4].

As currently available clinicopathological classification systems and treatment modalities fail to tailor patient management or improve survival substantially, molecular subtyping of PDAC may help unravel its mechanisms of carcinogenesis and progression, and help discover efficient therapeutic molecules. The quest to identify clinically relevant gene signatures of PDAC has been a rough journey resulting in a wide range of often non-reproducible or conflicting data. Recently, based on 27 microdissected surgical samples, three subtypes of PDAC (classical, quasimesenchymal, and exocrine-like) were identified and their gene signatures defined as *PDAssign*. Despite its small sample size the study presented a prognostic relevance for these subtypes [5]. The aim of our study was to evaluate the prognostic relevance of molecular subtypes and identify key transcription factors as master regulators in a large cohort of PDAC patients. Hereto, in contrast to other studies, we analyzed also several relevant clinicopathological variables that have proven to influence survival significantly.

## Methods

### Data collection

Between 1998 and 2010, tissue samples were collected, after written informed consent, from patients who underwent pancreatic resection for PDAC. Snap-frozen tissue samples were stored in liquid nitrogen and/or at −80 °C in RNALater (Qiagen) until further use. From the primary tumor of 171 patients and from surrounding non-tumoral pancreatic (control) tissue of 14 patients, total RNA was extracted using the RNeasy Mini kit (Qiagen) according the manufacturer’s instructions. Only samples with an RNA integrity number (RIN) of >7.0 were used for further analysis, i.e. 118 PDAC samples (male/female ratio: 65/53; age: 32–87 years with median of 64 years) and 13 control tissues (male/female ratio: 8/5; age: 51–78 years with median of 67 years). Two pathologists confirmed PDAC samples to contain at least 30 % cancer cells. Patients with pre-operative radio- or chemotherapy were excluded from the study.

### Microarray hybridization

RNA concentration and purity were determined spectrophotometrically using the Nanodrop ND-1000 (Nanodrop Technologies) and RNA integrity was assessed using a Bioanalyser 2100 (Agilent). Per sample, an amount of 100 ng of total RNA spiked with bacterial RNA transcript positive controls (Affymetrix) was amplified and labeled using the GeneChip 3′ IVT express kit (Affymetrix). All steps were carried out according to the manufacturers protocol (Affymetrix). A mixture of purified and fragmented biotinylated amplified RNA (aRNA) and hybridisation controls (Affymetrix) was hybridized on Affymetrix Human Genome U219 Array Plate followed by staining and washing in the GeneTitan® Instrument (Affymetrix) according to the manufacturer’s procedures. To assess the raw probe signal intensities, chips were scanned using the GeneTitan® HT Array Plate Scanner (Affymetrix).

### Microarray data analysis

Analysis of the microarray data was performed with the Bioconductor/R packages [6] (http://www.bioconductor.org). The analysis was based on the Robust Multi-array Average (RMA) expression levels of the probe sets, computed with the package xps. Differential expression was assessed via the moderated t-statistic implemented in the limma package, described in [7]. To control the false discovery rate, multiple testing correction was performed [8] and probe sets with a corrected *p*-value below 0.05 and an absolute fold change larger than two were selected.

### Molecular subtype discovery

#### Gene filtering

Intrinsically variable genes were first selected based on their expression variation over the 118 PDAC samples (2374 genes with s.d. > 0.8). The “*PDAssign*” genes were selected as the variable genes matching the published signature [5], i.e. 62 genes excluding 3 genes without probes in our microarray platform (*CELA3B*, *PRSS2*, *SLC2A3*) and 3 genes that are not variable (*SLC16A1, GPM6B, SLC5A3*).

#### Identification of subclasses using non-negative matrix factorization clustering

Subclasses of a data set consisting of unified expression data of 118 samples and variable genes were computed by reducing the dimensionality of the expression data from thousands of genes to a few metagenes by applying a consensus non-negative matrix factorization (NMF) clustering method (v5) [9, 10]. This method computes multiple k-factor factorization decompositions of the expression matrix and evaluates the stability of the solutions using a cophenetic coefficient. Consensus matrices and sample correlation matrices were calculated for 2 to 5 potential subtypes (k) using default parameters and Euclidian distance. The final subclasses were defined based on the most stable k-factor decomposition and visual inspection of sample-by-sample correlation matrices. For this we used the NMF clustering implemented from Gene Pattern software package [11].

#### Merging microarray data using DWD

Distance Weighted Discrimination (DWD) method [12] was applied for batch correction to the data of Collisson et al. and our expression data on variable genes after row median centering and column normalization according to the authors’ protocol [5]. The Java version of DWD was used with default parameters (Standardized DWD, centered at zero).

### Bioinformatic analysis

Gene Set Enrichment Analysis (GSEA) was used to score how enriched the modules and regulons (identified above in the first section) were in the top differentially expressed genes for a given contrast [13]. We performed the GSEA Preranked analysis using the list of the genes ranked by the signed *p*-value from each of the supervised and unsupervised biological contrasts (e.g. PDAC vs Control, k2.cl1 vs k2.cl2). This algorithm scores the positive or negative enrichment for all modules/regulons at the top or the bottom of the ranking. We also used WebGestalt [14], in which the hyper-geometric test was used for enrichment analysis and the Benjamini-Hochberg procedure was used to control the False Discovery Rate.

Top 250 KRAS dependency signature probes were extracted from Singh et al. [15] and provided a list of 187 genes, of which 165 genes were in our microarray data and 77 genes showed variable expression (sd > 0.8). The list of 77 genes was ranked according to their KRAS dependency and was used to make an expression heatmap of the 118 PDAC samples. Expression heatmaps are generated using R package *heatmap*. Hierarchical clustering based on a Spearman rank correlation as distance metric and an average linkage method (R function *hclust*) was used predicting 112 samples (95 %) as KRAS dependent samples (high level KRas activity) and 6 samples as KRAS independent (low level KRAS activity). The R function *cutree* automatically cut each dendrogram (from the top down) to form two groups of samples. *KRAS* expression levels are also significantly higher in KRAS dependent samples compared to other samples (*p* = 0.002).

### Survival analysis

Kaplan-Meier estimates were used for survival analysis. Overall survival (OS) was defined as time from surgery to death, irrespective of cause. Disease-free survival (DFS) was defined as time to tumor recurrence or death, irrespective of cause. Patients were followed up until death or until the date of study closure on November 2014. Together with the molecular subclasses the effect on survival of a set of 16 clinico-pathological prognostic factors was evaluated: patient age (years), gender (male/female), PDAC location (head/body or tail), tumor diameter (mm), differentiation grade (pG), depth of tumor invasion (pT), locoregional lymph node metastasis (pN), distant organ metastasis (pM), completeness of tumor resection (pR), magnitude of the surgical resection margin (pRM), perineural invasion (PNI), vascular invasion (VI), lymph vessel invasion (LVI), extra-capsular lymph node invasion (ECLNI), AJCC TNM Classification 7th Edition, adjuvant systemic chemotherapy (Yes/No). Log-rank tests and Cox regression models were used to verify the relation between a set of predictors and survival. A multivariable model was constructed combining the predictors with *p* < 0.10 in the univariable models, and *p* values less than 0.05 were considered significant.

### Master regulator analysis

In order to characterize regulatory networks underlying the subtypes, we used *iRegulon* [16] to identify master regulators, i.e. transcription factors whose regulons (transcriptional target sets) are highly overlapping with the observed gene signatures. The master regulators are expected to be directly activated by signal transduction. In this approach, we use a large collection of transcription factor (TF) motifs (9713 motifs for 1191 TFs) and a large collection of ChIP-seq tracks (1120 tracks for 246 TFs).

Briefly, this method relies on a *ranking-and-recovery* strategy where the offline ranking aims at ranking 22284 genes of the human genome (hg19) scored by a motif discovery step integrating multiple cues, including the clustering of binding sites within *cis*-regulatory modules (CRMs), the potential conservation of CRMs across 10 vertebrate genomes, and the potential distal location of CRMs upstream or downstream of the transcription start site (TSS+/−10 kb). The recovery step calculates the TF enrichment for each set of genes, i.e. genes from co-expression modules, leading to the prediction of the TFs and their putative direct target genes in the module. An important advance of this method is that it can optimize the association of TFs to motifs using not only direct annotations, but also predictions of TF orthologs and motif similarity, allowing the discovery of more than 1191 TFs in human.

### HNF1B immunohistochemistry

Samples (*n* = 6) showing top differential expression for HNF1B were selected for HNF1B immunohistochemistry staining (IHC). Five-micrometer-thick sections were prepared from formalin-fixed paraffin-embedded PDAC specimens. Stainings were made using the Benchmark Ultra (Ventana). Briefly, samples were deparaffinized at 72 °C and endogenous peroxidase activity was blocked using 0.3 % H_2_O_2_. Antigens were retrieved by heating the sections for 68 min at 91 °C in citrate buffer, pH6. Sections were incubated with the primary antibody against human HNF1B (Sigma, catalogue number HPA002083) dissolved 1:200 in Dako REAL antibody diluent at 37 °C for 32 min. The reaction product was developed using ultraView Universal DAB Detection Kit and sections were counterstained with hematoxylin. Sections were washed, dehydrated in progressively increasing concentration of ethanol and xylene, and mounted with xylene-based mounting medium. Normal human pancreas was used as a positive control. In order to check unspecific antibody binding, negative controls, in which the primary antibody was omitted, were also done. Samples were carefully analyzed by a pathologist. Slides were visualized using Leica DMR microscope (Leica Microsystems Ltd, Germany) and photographs were taken using Leica Application Suite v3.5,0 software (Leica Microsystems, Switzerland). HNF1B staining was scored based on intensity (on a scale from 0–3; 0, negative; 1, weak; 2, positive; 3, strong) and the proportion of reactive cells (0–100 %); histoscore was determined by multiplying both parameters (range 0–300) as published in Hoskins et al. [17]. When more than one magnification area was available from a given tumor, the mean score was used.

## Results

### Gene expression profiling

We applied gene expression profiling using microarrays on 118 tumor and 13 histologically normal pancreatic tissue samples (control) to investigate the molecular mechanisms driving PDAC and its different subtypes. Gene expression analysis of PDAC samples was performed on whole tumor tissue, i.e. cancer cells (at least 30 % of sample) and tumor stroma. Differential gene expression analysis using the contrast of all PDAC samples versus all control samples provided a large number (*n* = 6873) of genes that were differentially expressed (corrected *p*-value < 0.05; Additional file 1: Table S1). Our findings are in agreement with previously published pancreatic cancer gene expression data [18]. When we compared the gene expression profile of each tumor sample against a published KRas dependent gene signature [15], we found 94 % of our samples (112/118) to be KRas-dependent, which is in agreement with the fact that more than 90 % of PDAC have a *KRAS* driver mutation (Additional file 2: Figure S1) [19, 20].

### Molecular subtypes linked to survival

**Fig. 1:**
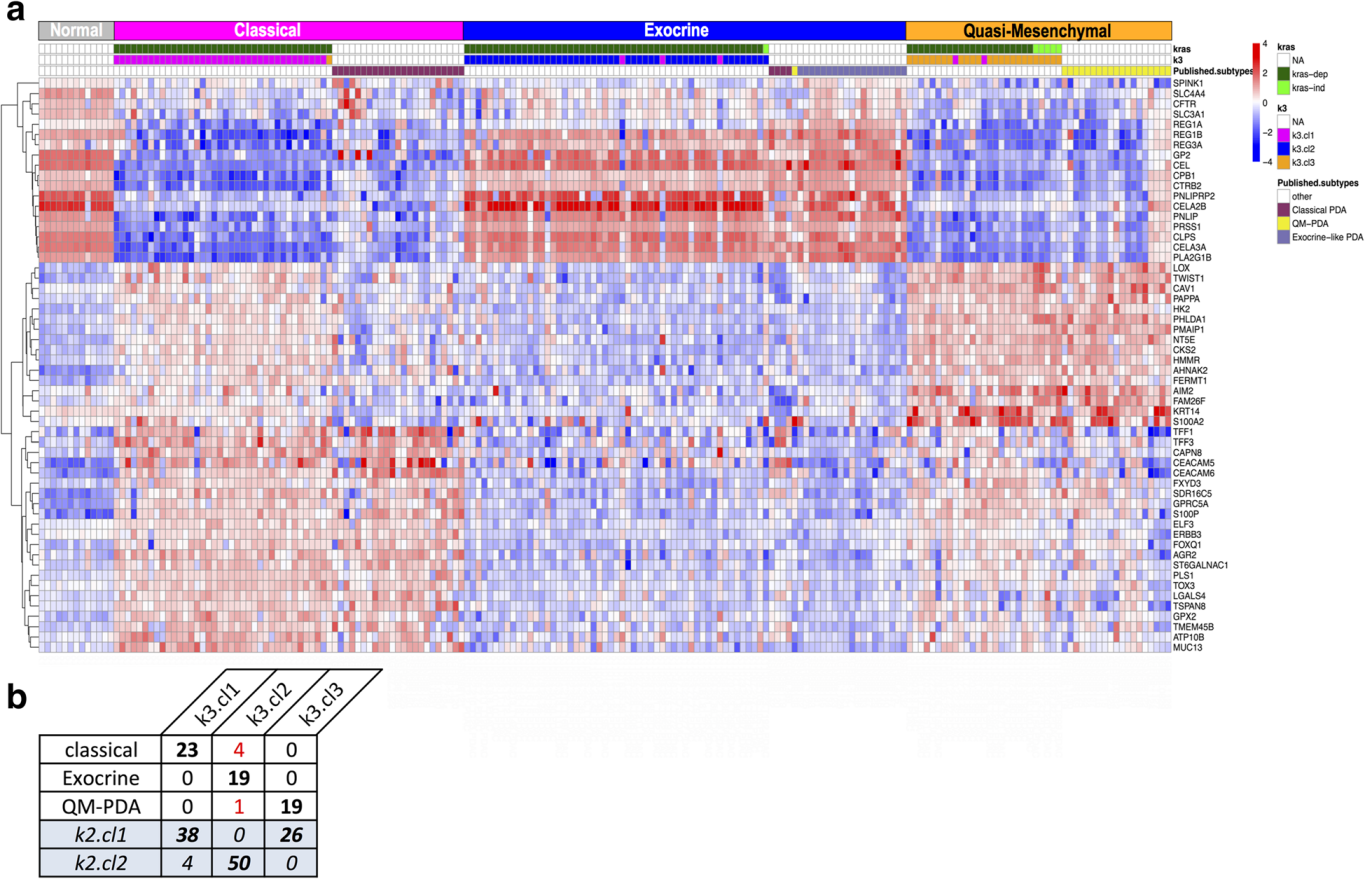
Expression heatmap for merged data. **a** Heatmap for 56 *PDAssign* genes vs 184 PDAC samples (+13 histologically normal pancreatic tissue samples as “Control” samples in *grey*). Samples are ordered and clustered by NMF clusters obtained from the NMF clustering of the merged PDAC data. Genes are clustered by hierarchical clustering using Pearson correlation distance (complete linkage). Sample legends show the sample clustering of the published subtypes (for the UCSF and GSE15471 tumors), but also the different predicted clusters from NMF of our 118 PDAC data (k3) and the predicted K-Ras dependency (kras) (see also Additional file 2: Figure S1 and Additional file 3: Figure S2). **b** Comparison of the predicted subtypes and known subtypes at the sample levels

Recently, Collisson et al. studied gene expression profiles of 27 microdissected PDAC samples, and identified three molecular subtypes that are driven by the 62-gene *PDAssign* signature, namely a classical, quasi-mesenchymal, and exocrine-like subtype. These three subtypes were found significantly linked to survival. The classical subtype was associated with the best survival, whereas the quasi-mesenchymal subtype with the worst survival [5]. We used the *PDAssign* to classify our 118 PDAC samples using NMF clustering, whereby the number of clusters/subtypes (k) is a parameter. When k is set to 2, 3, 4, or 5, the analyses resulted in a stable clustering for (all have cophenetic coefficient > 0.99) (Additional file 3: Figure S2a). When we merged our data with those of Collisson et al., we found almost a perfect match (92.4 %) with their subtypes (Fig. 1). This finding cross-validates the *PDAssign* signature on a large dataset of whole-tumor samples with high-quality RNA.

**Fig. 2:**
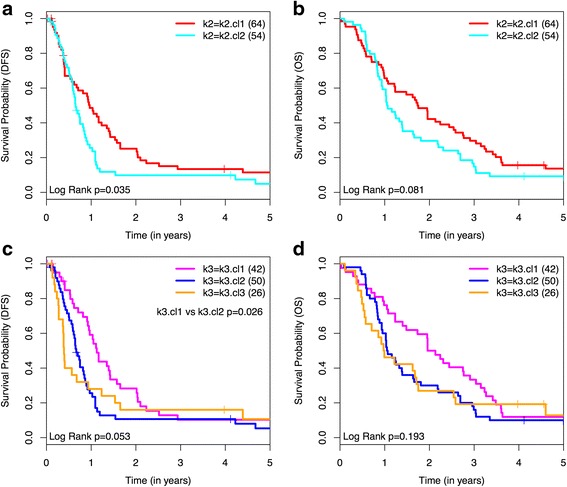
Disease-free (DFS) and overall survival (OS) of patients according to molecular subtypes of PDAC. Molecular subtypes are predicted by using the published *PDassign* genes as a classifier of our PDAC samples. Survival according to 2 molecular subtypes (k2) classification: **a** DFS is significantly better for k2.cl1 (*red* line) than that for k2.cl2 (*blue* line) (*p* = 0.035). **b** No statistically significant difference in OS is observed between k2.cl1 (*red* line) vs. k2.cl2 (*blue* line) (*p* = 0.081). Survival according to 3 molecular subtypes (k3) classification: **c** DFS is significantly better for k3.cl1 (*magenta* line) than that for k3.cl2 (*blue* line) (*p* = 0.026). **d** No statistically significant difference in OS is observed between the 3 subtypes separately (*p* = 0.193); k3.cl1 (*magenta* line), k3.cl2 (*blue* line), k3.cl3 (*orange* line). Tables 1 and 2 provide more information on these survival curves

We also confirmed the association of the classical subtype (k3.cl1) with the best survival (DFS and OS) as compared to the other subtypes (Fig. 2). For the exocrine-like (k3.cl2) subtype, Collisson et al. provided an intermediate survival profile, though this was based on survival data from 5 patients only. Our results from 50 exocrine-like subtype PDAC patients showed the exocrine-like subtype to be associated with worse survival than the classical subtype, and comparable to that of the quasi-mesenchymal (k3.cl3) subtype.

The results of the univariable and multivariable models for OS and DFS are listed in Tables 1 and 2. Univariable analyses identified several variables affecting either OS or DFS. In multivariable analyses molecular subtype k2 was the only independent predictor of both OS (*p* = 0.031) and DFS (*p* = 0.034). Other independent predictors of OS were molecular subtype k3 (*p* = 0.017) and age (*p* = 0.008). In other words, we could use the gene expression of the *PDAssign* signature to classify new patient samples into one of three subtypes (using k3), or one of two subtypes (using k2) and predict a link to survival. Note that for k2, almost all the samples (92 %, 50/54) of the exocrine subtype remain as a separate group, while the second cluster, k2.cl1, unites the classical and QM subtypes together. These results suggest that molecular subtypes, but not clinicopathological factors, can be used as independent predictors of survival.

**Table 1:** Results of univariable and multivariable Cox regression models for disease-free survival (DFS)

		Number of Patients	Disease-free Survival Time (DFS; median (CI): months)	Univariable		Multivariable	
				Hazard ratio (HR) (95% CI)	*p*-value	Hazard ratio (HR) (95% CI)	*p*-value
Clinicopathological Parameter							
Age	< 64 y.	58	10.2 (6.4 - 13.3)	0.756 (0.508 - 1.122)	0.165		
	> 64 y.	60	9.0 (7.3 - 10.9)				
Gender	Female	53	10.9 (8.1 - 13.5)	0.785 (0.528 - 1.161)	0.226		
	Male	65	7.8 (6.0 - 11.1)				
PDAC Location	Head	93	9.8 (7.4 - 12.4)	0.564 (0.357 - 0.921)	**0.023**	0.581 (0.340 - 1.015)	0.056
	Body or Tail	25	8.3 (4.4 - 11.1)				
Tumor diameter	< 2 cm	27	13.1 (6.7 - 17.0)	0.676 (0.411 - 1.064)	0.092	0.739 (0.399 - 1.306)	0.306
	> 2 cm	91	7.8 (3.3 - 18.9)				
pG	1	10	13.1 (4.0 - 24.3)		0.715		
	2	41	10.2 (7.1 - 16.2)				
	3	67	7.7 (6.0 - 11.0)				
pT	1	2	7.7 (7.3 - 8.1)		0.857		
	2	13	11.6 (7.1 - 17.1)				
	3	95	9.0 (6.8 - 11.4)				
	4	8	8.4 (7.4 - 11.2)				
pN	0	45	10.3 (6.1 - 13.1)	0.839 (0.557 - 1.248)	0.39		
	1	73	8.8 (7.3 - 11.4)				
pM	0	105	10.0 (7.7 - 12.3)	0.411 (0.230 - 0.802)	**0.011**	0.441 (0.097 - 1.458)	0.19
	1	13	4.7 (3.2 - 10.9)				
pR	0	91	10.0 (7.8 - 12.4)	0.712 (0.458 - 1.149)	0.159		
	1	27	7.7 (7.4 - 11.2)				
pRM	< 1 mm	64	10.0 (6.3 - 13.1)	0.925 (0.621 - 1.382)	0.7		
	> 1 mm	50	9.8 (7.0 - 12.3)				
PNI	0	15	17.1 (3.3 - 56.1)	0.528 (0.264 - 0.955)	**0.034**	0.516 (0.233 - 1.053)	0.07
	1	101	9.8 (7.4 - 11.4)				
VI	0	36	11.1 (7.0 - 18.9)	0.629 (0.399 - 0.969)	**0.035**	0.805 (0.485 - 1.303)	0.382
	1	76	8.0 (6.4 - 11.0)				
LVI	0	34	10.2 (6.1 - 14.3)	0.833 (0.529 - 1.276)	0.408		
	1	81	8.8 (7.3 - 11.4)				
ECLNI	0	69	10.4 (7.3 - 13.3)	0.821 (0.544 - 1.254)	0.356		
	1	43	8.4 (6.3 - 11.1)				
AJCC TNM Stage 7th Ed.	≤ 2a	38	10.9 (6.8 - 14.3)	0.730 (0.474 - 1.100)	0.134		
	≥ 2b	80	8.5 (7.0 - 11.1)				
	N0 <T3 M0 (Early)	38	10.9 (6.8 - 14.3)	Early vs Adv 0.498 (0.273 - 0.950)	Overall 0.104	Overall	0.209
	N1 <T3 M0 (LNM)	62	9.8 (7.3 - 12.6)		Early vs Adv **0.035**	0.915 (0.295 - 4.022)	0.892
	T4 or M1 (Advanced)	18	5 (3.3 - 10.4)				
Adjuvant chemotherapy	0	36	7.4 (4.6 - 11.0)	1.139 (0.733 - 1.728)	0.553		
	1	82	10.0 (7.7 - 12.6)				
Molecular Subtypes							
k2	Cluster 1	64	11.6 (7.4 - 16.2)	0.655 (0.440 - 0.976)	**0.035**	0.252 (0.092 - 0.888)	**0.034**
	Cluster 2	54	7.8 (6.7 - 10.0)				
k3	Cluster 1	42	13.5 (10.9 - 17.1)	Cl1 vs Cl2 0.602 (0.382 - 0.940)	Overall 0.053	Overall	0.318
	Cluster 2	50	8.0 (7.0 - 10.0)	Cl1 vs Cl3 0.615 (0.363 - 1.066)	Cl1 vs Cl2 **0.026**		
	Cluster 3	26	4.7 (3.3 - 11.2)		Cl1 vs Cl3 0.082		
k4	Cluster 1	39	13.3 (9.8 - 16.7)	Cl1 vs Cl2 0.670 (0.418 - 1.065)	Overall 0.333	Overall	0.751
	Cluster 2	45	8.4 (6.7 - 10.0)		Cl1 vs Cl2 0.090		
	Cluster 3	7	11.0 (4.8 - 17.1)				
	Cluster 4	27	4.7 (3.3 - 11.2)				
k5	Cluster 1	41	13.5 (10.9 - 17.0)	Cl1 vs Cl5 0.488 (0.251 - 1.021)	Overall 0.209	Overall	0.616
	Cluster 2	35	8.4 (7.0 - 10.2)		Cl1 vs Cl5 0.057		
	Cluster 3	4	9.3 (4.8 - NA)				
	Cluster 4	26	4.7 (3.3 - 11.2)				
	Cluster 5	12	7.5 (4.3 - 11.0)				

Differences between variables or subgroups with a *p*-value of > 0.1 are not shown in the table and bold fonts indicate significant values (<0.05)

**Table 2:** Results of univariable and multivariable Cox regression models for overall survival (OS)

		Number of patients	Overall survival time (OS; median (CI): months)	Univariable		Multivariable	
				Hazard ratio (HR) (95% CI)	*p*-value	Hazard ratio (HR) (95% CI)	*p*-value
Clinicopathological parameter							
Age	< 64 y.	58	23.5 (12.6 - 33.0)	0.626 (0.422 - 0.924)	**0.018**	0.551 (0.350 - 0.859)	**0.008**
	> 64 y.	60	13.7 (11.4 - 16.8)				
Gender	Female	53	20.5 (12.9 - 29.3)	0.865 (0.587 - 1.269)	0.459		
	Male	65	12.6 (11.2 - 20.1)				
PDAC location	Head	93	19.5 (12.6 - 23.5)	0.600 (0.384 - 0.967)	**0.036**	0.714 (0.435 - 1.209)	0.204
	Body or Tail	25	12.6 (10.0 - 26.4)				
Tumor diameter	< 2 cm	27	20.5 (11.7 - 36.5)	0.746 (0.459 - 1.165)	0.204		
	> 2 cm	91	14.8 (11.9 - 20.8)				
pG	1	10	22.5 (1.5 - 33.2)		0.89		
	2	41	14.8 (11.2 - 29.3)				
	3	67	15.9 (11.5 - 23.5)				
pT	1	2	13.4 (12.3 - 14.6)		0.694		
	2	13	26.9 (9.4 - 38.8)				
	3	95	15.9 (11.8 - 21.0)				
	4	8	12.4 (1.3 - 33.0)				
pN	0	45	21.0 (14.6 - 29.3)	0.750 (0.499 - 1.111)	0.154		
	1	73	12.8 (11.7 - 17.8)				
pM	0	105	17.8 (12.9 - 23.5)	0.569 (0.323 - 1.097)	0.089	0.624 (0.166 - 1.928)	0.427
	1	13	11.4 (5.8 - 12.4)				
pR	0	91	16.8 (12.9 - 25.6)	0.733 (0.474 - 1.174)	0.19		
	1	27	12.4 (7.0 - 23.4)				
pRM	< 1 mm	64	15.4 (11.8 - 25.6)	1.096 (0.737 - 1.622)	0.647		
	> 1 mm	50	16.7 (12.3 - 23.5)				
PNI	0	15	37.8 (10.6 - NA)	0.468 (0.227 - 0.860)	**0.013**	0.561 (0.252 - 1.115)	0.103
	1	101	15.9 (12.4 - 20.8)				
VI	0	36	19.7 (11.9 - 33.2)	0.730 (0.466 - 1.115)	0.148		
	1	76	12.8 (11.5 - 23.4)				
LVI	0	34	19.7 (10.6 - 33.0)	0.877 (0.561 - 1.334)	0.547		
	1	81	15 (12.3 - 21.7)				
ECLNI	0	69	20.1 (12.9 - 30.5)	0.654 (0.437 - 0.987)	**0.043**	0.660 (0.398 - 1.089)	0.104
	1	43	12.4 (10.2 - 20.8)				
AJCC TNM Stage 7th Ed.	≤ 2a	38	23.5 (16.8 - 31.7)	0.681 (0.443 - 1.023)	0.065	0.672 (0.166 - 2.254)	0.53
	≥ 2b	80	12.6 (11.4 - 16.7)				
	Early (pN=0,pT≤ 3,pM=0)	38	23.5 (16.8 - 31.7)	Early vs LNM 0.722 (0.463 - 1.106)	Overall 0.105		
	pN=1,pT≤ 3,pM=0	62	14.8 (11.2 - 21.7)	LNM vs Adv 0.736 (0.425 - 1.328)	Early vs LNM 0.136		
	Advanced (pT=4 or pM=1)	18	11.7 (6.6 - 12.4)	Early vs Adv 0.532 (0.295 - 0.997)	Early vs Adv **0.049**		
Adjuvant chemotherapy	0	36	12.1 (7.0 - 16.8)	1.337 (0.874 - 2.002)	0.176		
	1	82	19.8 (13.9 - 25.6)				
Molecular subtypes							
k2	Cluster 1	64	20.9 (12.9 - 29.3)	0.710 (0.482 - 1.048)	0.081	0.247 (0.092 - 0.860)	**0.031**
	Cluster 2	54	12.7 (11.2 - 16.7)				
k3	Cluster 1	42	24.6 (16.8 - 33.2)	Cl1 vs Cl2 0.680 (0.437 - 1.050)	Overall 0.193	Overall	**0.017**
	Cluster 2	50	12.7 (11.2 - 16.7)	Cl2 vs Cl3 1.055 (0.641 - 1.788)	Cl1 vs Cl2 0.082		
	Cluster 3	26	11.8 (6.6 - 20.5)	Cl1 vs Cl3 0.717 (0.426 - 1.236)	Cl1 vs Cl3 0.226	Cl1 vs Cl3 0.209 (0.057 - 0.809)	**0.024**
k4	Cluster 1	39	23.5 (14.8 - 33.0)		0.577		
	Cluster 2	45	12.6 (10.6 - 16.7)				
	Cluster 3	7	26.2 (9.3 - 38.8)				
	Cluster 4	27	11.9 (6.6 - 21.0)				
k5	Cluster 1	41	23.5 (14.8 - 33.2)	Cl1 vs Cl5 0.398 (0.210 - 0.808)	Overall 0.122	Overall	0.271
	Cluster 2	35	13.9 (11.4 - 21.7)	Cl2 vs Cl5 0.483 (0.251 - 0.988)	Cl1 vs Cl5 **0.012**		
	Cluster 3	4	28.9 (9.3 - NA)	Cl3 vs Cl5 0.298 (0.067 - 0.949)	Cl2 vs Cl5 **0.046**		
	Cluster 4	26	11.8 (6.6 - 20.5)		Cl3 vs Cl5 **0.040**		
	Cluster 5	12	10 (6.9 - 16.6)				

Differences between variables or subgroups with a *p*-value of > 0.1 are not shown in the table and bold fonts indicate significant values (<0.05)

### Functional analysis of molecular subtypes

PDAC subtypes are poorly characterized at the molecular level and little is known about the regulatory networks underlying the expression of the genes driving better or worse survival. As we could reproduce the three subtypes (NMF with k = 3, or briefly “k3”) and confirmed their prognostic relevance, we aimed to further characterize their gene expression profiles, functions, and pathways. Compared to normal tissue samples, all subtypes are enriched for “Neoplasms”, “invasiveness”, and “integrin family cell surface interactions”, and all subtypes are comparably enriched for typical pancreatic cancer gene signatures (FDR = 0.000, NES > =2.41).

When the k3 subtypes are compared directly against each other (Additional file 1: Table S1), we could define cluster-specific gene signatures as the genes that are specifically over- or under-expressed for a given subtype and missing *PDAssign* genes were added to these signatures to perform functional enrichment analysis (Additional file 4: Figure S3). For example, we found a specific gene signature with 148 genes over-expressed and 3 under-expressed in the predicted exocrine-like subtype that is enriched for processes related to the exocrine pancreas, such as pancreatic secretion and protease activity. For the QM subtype we identified 50 up-regulated genes specific for this subtype with 132 down-regulated genes, and this set of genes shows typical properties of epithelial and mesenchymal cancers. Focusing further on Epithelial-to-Mesenchymal Transition (EMT) properties, we found an enrichment of an EMT signature (NES = 2.38). Some EMT TFs, such as *TWIST1* and *SNAI2*, show QM subtype specific expression. However, although this signature resembles some aspects of EMT, it does not capture the entire EMT signature, since there is limited gene overlap with a core mesenchymal transition signature derived by meta-analysis across cancer types [21]. Notice that samples clustered by low and high expression of mesenchymal cancer attractors do not show a significant link with survival. Finally, the predicted classical subtype has very few specific genes compared to the other subtypes (only 14 genes), and lacks any specific biological pathway enrichment. Overall, despite a partial gene overlap with the published *PDAssign* genes (36.4 %, 20/55) (Additional file 4: Figure S3e), our larger cluster-specific gene signatures agree with the known description of the PDAC subtypes.

### Master regulators of PDAC

**Fig. 3:**
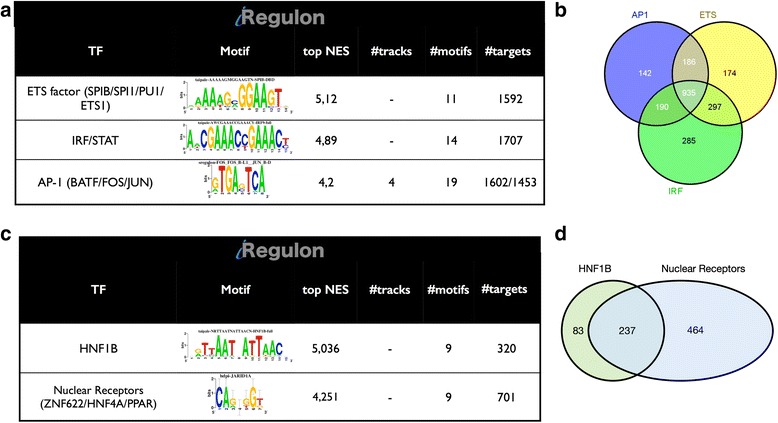
Master regulators in PDAC vs Control (histologically normal pancreatic tissue samples). **a** Result summary of the regulatory analysis with *iRegulon* on 2640 up regulated genes. **b** Venn diagram of the predicted up-regulated targets from AP1, ETS and IRF. **c** Results of the regulatory analysis with *iRegulon* on 1325 down-regulated genes. **d** Venn diagram of the predicted down-regulated targets from HNF1A/B and Nuclear Receptors. Raw results of the analysis are presented in Additional file 4: Table S3 and Additional file 5: Figure S4

In the set of 2640 up-regulated genes in PDAC versus Control, one of the most strongly enriched TF motifs were those for IRF/STAT with a normalized enrichment score (NES) of 4.89. We identified 1707 (64.5 %) of these genes as targets of IRF/STAT (Fig. 3a-b). To identify the most likely TFs that could bind to these motifs or target genes, we compared the expression profile of all IRF and STAT family members to the expression profile of the predicted target genes, across the entire PDAC cohort. Among all candidates, STAT1 and IRF9 showed the highest correlation with the mean expression profile of the specific predicted targets (Pearson correlation = 0.70 and 0.69, respectively; *p*-value < 2.2 × 10^−16^). Interestingly, both TFs IRF9 and STAT1 physically interact and cooperate in the same signaling pathways [22]. Note that the IRF/STAT network is not differentially active between the PDA subtypes, but rather shows high expression across all PDAC samples, compared to normal tissue samples (Additional file 5: Figure S4a-c). Several additional motifs for relevant TFs were highly enriched in the PDAC vs Control signature, such as motifs corresponding to ETS-domain transcription factors (*ETS1*, *SPIB*, *SPI1* and *PU.1*) and AP-1 motifs (Fig. 3a).

We also found a ZEB1 motif (NES = 3.91) in the regulatory analysis of 1325 down-regulated genes (Fig. 3c; clustered with “LMO2” motifs) while ZEB1 is up-regulated in PDAC samples (log ratio = 2.17, *p*-value = 2.32 × 10^−21^). This finding is consistent with ZEB1 being a repressor [23]. Expression of ZEB1 has been shown recently to be a strong predictor of survival in PDAC [24] and is a known TF inducing epithelial-mesenchymal transition (EMT) in cancer cells. Finally, we identified enriched GATA3 ENCODE tracks in the “classical” and “QM-PDA” specific gene signatures (NES ~ 4), but not in the contrasts of PDAC vs control (data not shown). Thus, besides the role of GATA6 in QM-PDA, as proposed by Collison et al., our data also suggests that GATA3 may be functional in the two other subtypes.

Within the set of 1325 down-regulated genes in PDAC versus control, the most strongly enriched TF motifs were those for HNF1A/B (NES = 5.036, Fig. 3c). The HNF1A/B regulon, defined by 320 predicted target genes, is furthermore differentially expressed between classical and QM subtypes (Additional file 5: Figure S4). HNF1A/B is also found as top enriched regulator (NES = 8.156) when using a gene signature specific for the classical subtype compared to the exocrine subtype (data not shown). Compared to HNF1A, HNF1B is the best candidate to bind to this motif because the *HNF1B* gene itself is also down-regulated in the tumor samples (log ratio = −1.34, *p*-value = 6.24 × 10^−5^) and its expression profile is strongly correlated with the predicted targets (Pearson correlation = 0.71, *p*-value < 2.2 × 10^−16^), although *HFN1A* is also strongly correlated with these genes (Pearson correlation = 0.52, *p*-value = 1.67 × 10^−10^).

### HNF1B protein expression in PDAC

**Fig. 4:**
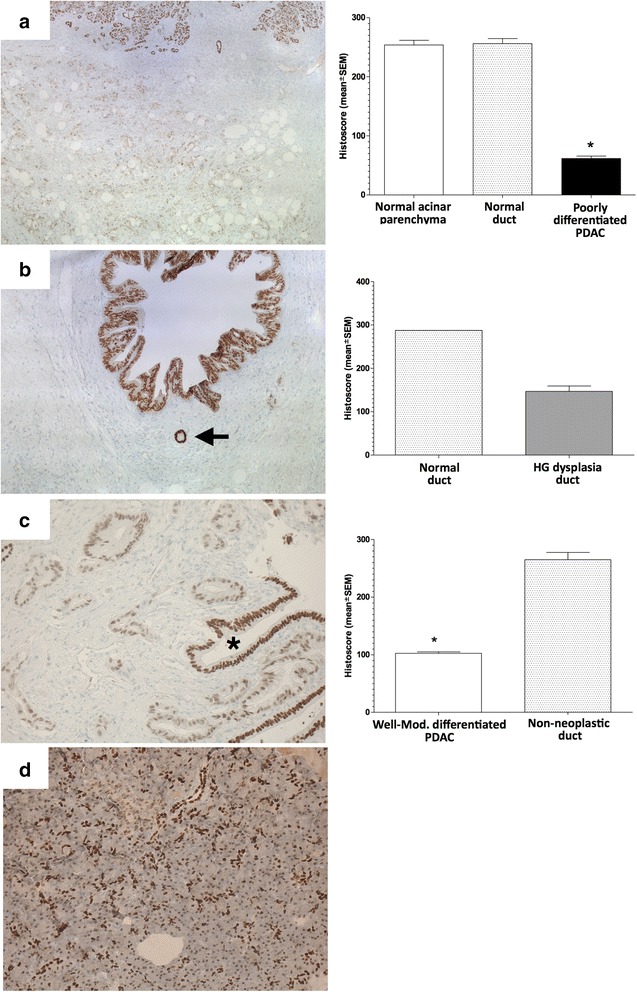
Immunohistochemistry for HNF1β. **a** Strong nuclear expression in normal acinar parenchyma and normal ducts (*upper* part) while the expression is completely lost in a poorly differentiated PDAC (*lower* part) (Magnification 50x). **b** IHC shows a lower expression in high-grade dysplasia (*upper* part) compared to normal duct (arrow) (Magnification 100x). **c** IHC for HNF1β shows reduced expression in a well to moderately differentiated PDAC compared to a non-neoplastic duct (asterisk) (Magnification 200x). Histograms showing the histoscores corresponding to the left (**a**) (**b**) (**c**). Asterisk on the histogram indicates that the differences with each of the other categories are significant (Mann Whitney test, *p* < = 0.0294). **d** Normal pancreas (positive control) showing a strong staining in ducts and in the acinar parenchyma (Magnification 40x)

As we identified HNF1B to be the strongest master regulator (NES = 5.036), we studied the expression of HNF1β on the protein level using immunohistochemistry (IHC) staining in normal and PDAC tumor samples. HNF1β is known as a marker of prostate [25, 26] and ovarian cancer [27, 28] but not of PDAC. HNF1B is also involved in endocrine pancreas development and in mesonephric duct formation [29]. IHC for HNF1β showed a clear nuclear staining (Fig. 4). We observed high expression levels of HNF1β in the acinar parenchyma (histoscore: mean ± SEM: 253, 7 ± 7, 8) and the ductal cells of normal pancreatic tissue (histoscore: 256, 0 ± 8, 5), while the connective tissue was negative. In premalignant lesions (high grade dysplasia), the expression was lower compared to normal ducts (histoscore = 287.5). A gradual loss of nuclear *HNF1β* expression was seen in well differentiated towards moderately and poorly differentiated tumors (histoscore: 102, 5 ± 2, 5 and 61, 8 ± 3,9) compared to a non-neoplastic duct (histoscore: 264, 9 ± 12, 7). Additionally, we screened nine human PDAC cell lines for the presence of HNF1B by IHC. We found, consistent with the gene expression analysis, that most malign pancreatic cell lines were negatives for HNF1B (Additional file 1: Table S2). Only one cell line (non-metastatic clone of SUIT2.028) was positive for HNF1B, while the highly metastatic clone (SUIT2.007) stay negative. Therefore, a loss or mutation of this gene might induce cancer. Since *HNF1B* is highly expressed in normal pancreatic ductal cells and loses its expression at that level in PDAC, HNF1β might represent a key player in PDAC carcinogenesis and progression.

## Discussion

In a recent attempt to unravel the tumor biology of pancreatic ductal adenocarcinoma (PDAC), Collisson et al. reported the *PDAssign* gene signature to classify this lethal cancer into three molecular subtypes with prognostic relevance [5]. The association of *PDAssign* with survival was based on gene expression data for 27 patients. In the current study, we evaluated the validity of *PDAssign* in a large cohort of 118 pancreatic cancer patients treated with surgery with or without adjuvant systemic chemotherapy. Apart from the sample sizes, another major difference between these two studies is the fact that we used whole-tumor samples including the micro-environment, whereas the former study used microdissection to enrich their samples for cancer cells. While microdissection of cells in fixed tissue could possibly be associated with higher levels of RNA degradation [30], we used high-quality samples with a pathologically proven minimum of 30 % cancer cells. By doing so, we kept the molecular information of the microenvironment, we have reduced RNA contamination and the large number of samples improves the signal-to-noise ratio. A future perspective may be to decipher the tumour specific response using single cell technology. Interestingly, a recent study shows that we can defined stroma and tumour specific subtypes by applying a similar NMF approach on a compendium of microarray expression data including 145 primary and 61 metastatic PDAC tumour samples [31]. They also identify two stromal subtypes, normal and activated, with the latter showing the worse prognosis. Nonetheless, we could confirm *PDAssign* to be a reliable classifier of PDAC into three distinct molecular subtypes with prognostic relevance. With their approach, Moffit et al. [31] virtually dissected the samples to identify a ‘classical’ and a ‘basal-like’ tumor-specific subtypes showing similarities to our predicted clusters with the ‘basal-like’ subtype showing genes of the same family of the ‘quasi-mesenchymal’ subtype with the worse survival. In our survival analysis, the clinicopathological factors are not independent predictors of survival, including stage and grade features. This is consistent with the recent studies [5, 31] but not with the previous literature [32], which may be due to the larger size of the recent studies. Moreover, we found these molecular subtypes as independent predictors of both disease-free and overall survival. We confirmed the classical PDAC subtype to be associated with the best survival, though in contrast to Collisson et al., we showed the exocrine-like subtype to be associated with poor prognosis and comparable survival to that of the quasi-mesenchymal subtype. We therefore envision that future evaluation of these molecular subtypes in larger studies may provide new insights in novel treatment strategies, opening new perspectives in personalized targeted therapy for PDAC.

In order to better understand the biology of PDAC we used *iRegulon* to identify transcription factors as master regulators of PDAC and its subtypes. We found that HNF1A/B are among thousand TFs the top enriched master regulators of the genes expressed in the normal pancreatic tissue compared to the PDAC regulatory network. On immunohistochemistry staining of PDAC samples we confirmed low expression of *HNF1B* in well differentiated tumors and no expression in six poorly differentiated PDAC samples. Our IHC results are also confirmed in an independent study from Jiang X et al. [33], i.e. positive staining for HNF1β in acinar parenchyma and ducts from normal pancreas and negative or moderate staining for PDAC samples. HNF1β also plays an important role in human normal pancreas morphogenesis and terminal differentiation of pancreatic β-cells [29]. Moreover, HNF1β is involved in regulating the β-cell transcription factor network and is necessary for glucose sensing or glycolytic signalling in the pancreatic β-cells [34]. *HNF1B* was also found recently to be down-regulated *in vitro* in PDAC cells by a microRNA mechanism involving hsa-miR-24 and/or hsa-miR-23a [35]. In the suggested mechanism, *HNF1B* deregulation in PDAC results in loss of the expression of the adhesion molecule E-cadherin, which induces epithelial-mesenchymal transition (EMT) and allows cells to detach from cell agglomerations and to migrate. In another study [36], *HNF1B* was also found deregulated in a mouse model of intraductal papillary mucinous neoplasm (IPMN) to PDAC progression with another duct-specific factor, *SOX9*, while the latter was not found deregulated in our data and was not predicted in our regulatory analysis. The authors highlighted the importance of these factors for the loss of mature ductal identity in tumor initiation. However, *HNF1A* has also been revealed as a specific key regulator of the transcriptome in pancreatic tumor tissues and was suggested as an important tumor suppressor in the pancreas [17]. Hoskins et al. observed that inducible over-expression of *HNF1A* in pancreatic tumor-derived cells could generate growth inhibition, a G0/G1 cell cycle arrest and apoptosis. Taken together, these observations suggest that HNF1A and HNF1B can be co-expressed in normal pancreatic tissues and may act as tumor suppressors through their regulatory activity. These factors can dimerize as homo- or hetero-dimers and can present several tissue-specific and species-specific isoforms [37], which can explain why we can find their activity independently. Low expression of *HNF1B* in some PDAC samples could reflect the tumor origination from acinar cells with incomplete ductal reprogramming phenotype (as suggested by one of our peer reviewer). Additionally, we identified IRF/STAT, AP-1, and ETS-family members as key transcription factors in gene signatures downstream of mutated KRAS. However, this approach only captures a part of the regulatory network while the post-transcriptional regulation and the microRNA regulatory network were not taken into account. We believe these key TFs or master regulators represent a valuable set of molecules for further study in functional assays and in vivo experiments to assess their role in PDAC carcinogenesis, progression, and novel therapeutic strategies.

## Conclusions

This is the first study describing in a large cohort of pancreatic cancer patients the prognostic relevance of molecular subtypes, which are driven by the *PDAssign* gene signature. Our results show molecular subtypes, but not clinicopathological factors, as independent predictors of survival. We have identified enriched transcription factors (TFs) as putative master regulators of PDAC, and their downstream networks, using *iRegulon*. Among them, the hepatocyte nuclear factor 1 homeobox A/B (HNF1A or HNF1B) and its predicted targets are globally down-regulated in PDAC. Immunohistochemistry for HNF1B shows a strong nuclear staining of normal pancreatic ductal cells, whereas its expression is low in malignant ductal cells of well differentiated and absent in poorly differentiated PDAC samples. As these TFs play a key role in PDAC, they may involve novel therapeutic targets to improve the survival of patients with PDAC.

## Abbreviations

CRM, *cis*-regulatory module; DFS, disease-free survival; DWD, distance weighted discrimination; EMT, epithelial-to-mesenchymal transition; GSEA, gene set enrichment analysis; HNF1A/B, hepatocyte nuclear factor 1 homeobox A/B; IHC, immunohistochemistry; NES, normalized enrichment score; NMF, non-negative matrix factorization; OS, overall survival; PDAC, pancreatic ductal adenocarcinoma; QM, quasi-mesenchymal; TF, transcription factor

## Additional files

Additional file 1:Supplementary Tables.
**Table S1.** Differential expression analysis performed by limma R package. **Table S2.** HNF1β expression in different human pancreatic cancer cell lines. **Table S3.** Master regulatory Results of gene set up-regulated in PDAC versus Control. The results are based on the enrichment score of the motifs (column 1), scores are the Area Under the Curve (AUC) and the Normalized Enrichment Score (NES). Motifs clusters are shown in Cluster code column. Predicted associated Transcription factors and predicted targets are shown in the two last columns. The TF view display by iRegulon integrates the results per clusterCode and prioritize the putative TF associated to a given cluster of motifs. **Table S4.** Master regulatory Results of gene set down-regulated in PDAC versus Control. See legend in Table S3. (XLSX 205 kb) Additional file 2: Figure S1. KRAS-dependence analysis. (a) Expression heatmap of the PDAC samples after hierarchical clustering of the samples for the 77 variable genes that overlap with the set of top 250 K-Ras dependence gene signature. The 6 samples predicted as KRAS independent samples are in the left (blue clusters vs green clusters). Genes are sorted according to the decreasing K-Ras dependence score. Levels of expression of KRAS gene signature (b) and of KRAS gene (c) in the different sample clusters. (d) Principal Component Analysis on the expression profiles of KRAS gene signature allows to cluster the samples into Control, KRAS dependent or KRAS independent samples (performed with R packages *prcomp* and *ggbiplot*). (TIFF 1478 kb) Additional file 3: Figure S2. Sample clustering using Non-negative Matrix Factorization (NMF). (a) Clustering of 118 PDAC samples with k = 2 to 7 using the normalized expression profiles of 59 *PDAssign* genes. The plot in the left shows the value of the cophenetic coefficient for different k values (k = 2 to 7) indicating the stability of the sample clustering. When we applied NMF clustering in an unsupervised approach (using all 2374 variable genes instead of the 56 *PDAssign* genes), the clustering of our samples into two, four or five subtypes are predicted to be more stable than three subtypes, although these are not associated with survival (b-c). (b) Clustering of 118 PDAC samples with k = 2 to 5 using the normalized expression profiles of variable genes (sd > 0.8). (c) Kaplan-Meier plots showing Overall Survival for the NMF predicted clusters presented in (b), i.e. molecular subtypes predicted by using the variable genes as a classifier of our PDAC samples instead of the *PDAssign* genes as shown in Fig. 2. Log Rank *p*-values are shown for the Disease Free Survival and Overall Survival in each plot. (TIFF 554 kb) Additional file 4: Figure S3. Subtype functional characterization. (a) Venn diagram for differentially expressed genes for the predicted subtype comparisons (limma, adjust.method = BH, padj.thr = 0.05, lfc.thr = 1). Expression heatmaps of up- and down-regulated genes in Exocrine subtype (b), QM-PDA subtype (c), and Classical subtype (d). Gene overlap between *PDAssign* genes and only genes specifically up-regulated in our predicted subtypes is shown in (e). Genes from the overlap are listed on each heatmap (b, c, d). (EPS 12073 kb) Additional file 5: Figure S4. Expression Levels of Master regulators and their targets identified in PDAC vs Control. Expression levels by predicted subtype of IRF predicted targets (a), IRF9 (b), STAT1 (c), HNF1B regulon (d), HNF1B probes (e) and HNF1A probes (f). (TIFF 1117 kb)
