# Supplementary figures and images for: Prognostic relevance of molecular subtypes and master regulators in pancreatic ductal adenocarcinoma
Source: BMC Cancer. 2016 Aug 12;16:632. doi: 10.1186/s12885-016-2540-6 (PMC4983037; doi:10.1186/s12885-016-2540-6)

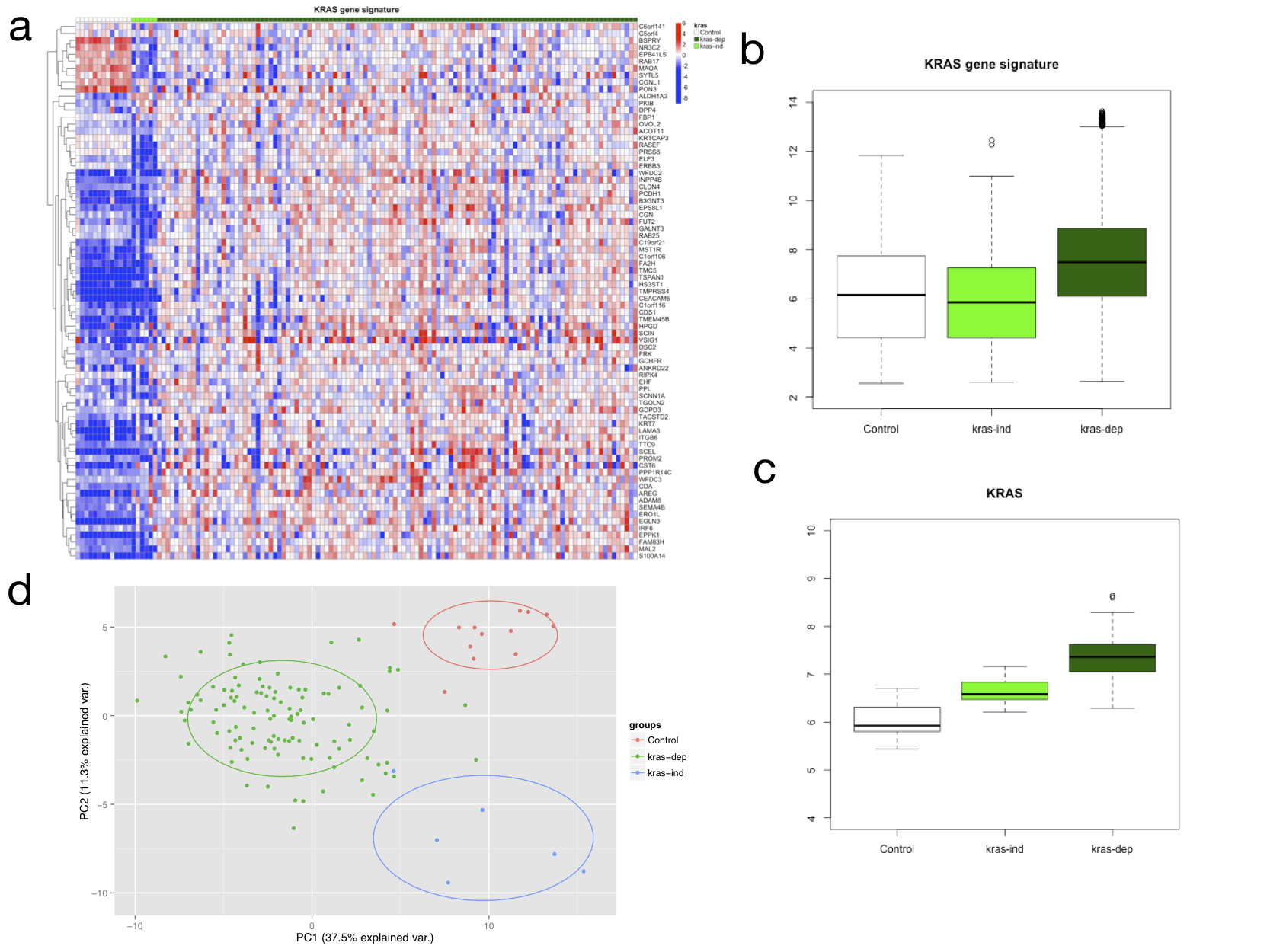

Supplement: Additional file 2: Figure S1. — KRAS-dependence analysis. (a) Expression heatmap of the PDAC samples after hierarchical clustering of the samples for the 77 variable genes that overlap with the set of top 250 K-Ras dependence gene signature. The 6 samples predicted as KRAS independent samples are in the left (blue clusters vs green clusters). Genes are sorted according to the decreasing K-Ras dependence score. Levels of expression of KRAS gene signature (b) and of KRAS gene (c) in the different sample clusters. (d) Principal Component Analysis on the expression profiles of KRAS gene signature allows to cluster the samples into Control, KRAS dependent or KRAS independent samples (performed with R packages prcomp and ggbiplot). (TIFF 1478 kb) [file 12885_2016_2540_MOESM2_ESM.tiff]

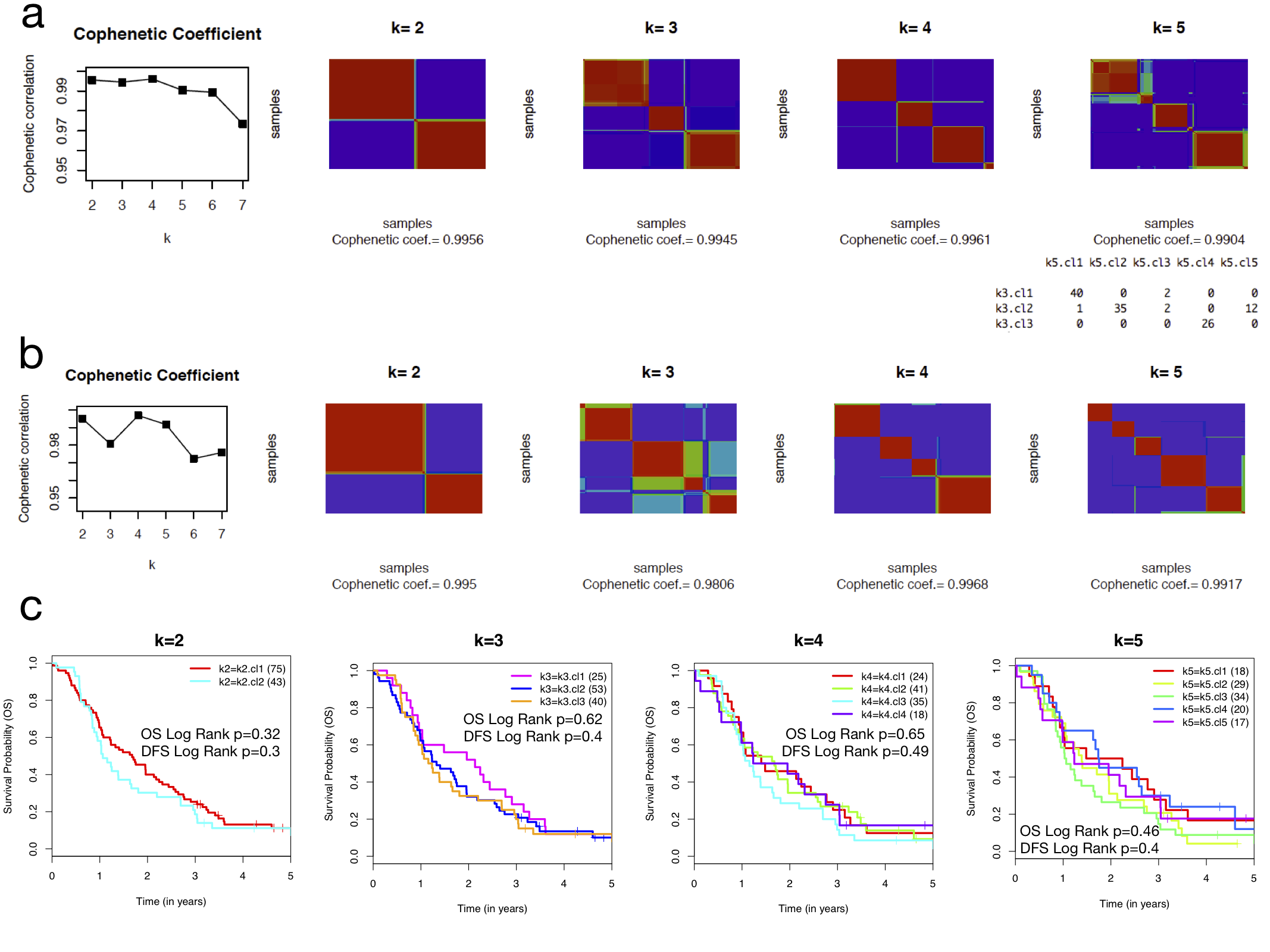

Supplement: Additional file 3: Figure S2. — Sample clustering using Non-negative Matrix Factorization (NMF). (a) Clustering of 118 PDAC samples with k = 2 to 7 using the normalized expression profiles of 59 PDAssign genes. The plot in the left shows the value of the cophenetic coefficient for different k values (k = 2 to 7) indicating the stability of the sample clustering. When we applied NMF clustering in an unsupervised approach (using all 2374 variable genes instead of the 56 PDAssign genes), the clustering of our samples into two, four or five subtypes are predicted to be more stable than three subtypes, although these are not associated with survival (b-c). (b) Clustering of 118 PDAC samples with k = 2 to 5 using the normalized expression profiles of variable genes (sd > 0.8). (c) Kaplan-Meier plots showing Overall Survival for the NMF predicted clusters presented in (b), i.e. molecular subtypes predicted by using the variable genes as a classifier of our PDAC samples instead of the PDAssign genes as shown in Fig. 2. Log Rank p-values are shown for the Disease Free Survival and Overall Survival in each plot. (TIFF 554 kb) [file 12885_2016_2540_MOESM3_ESM.tiff]

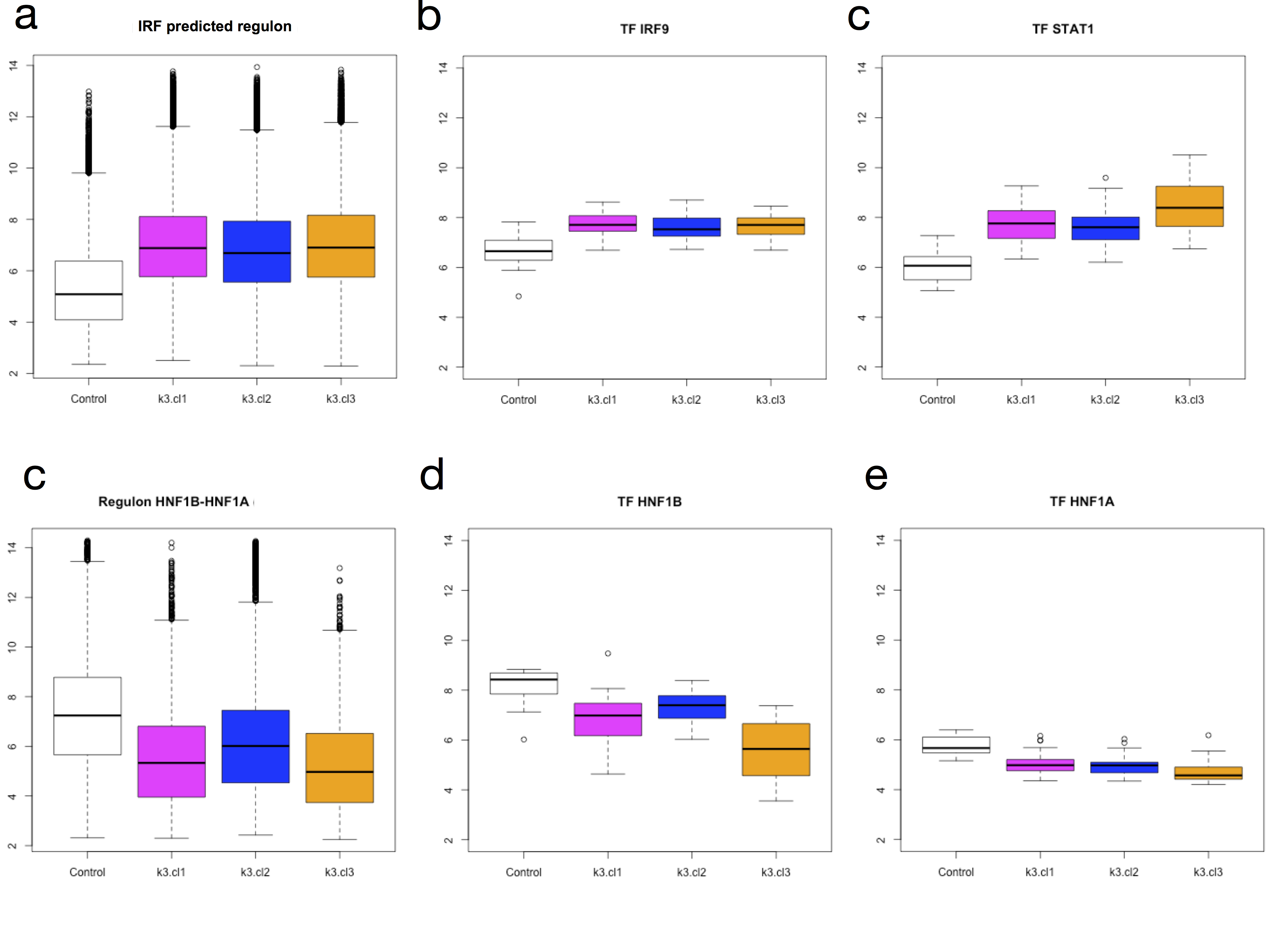

Supplement: Additional file 5: Figure S4. — Expression Levels of Master regulators and their targets identified in PDAC vs Control. Expression levels by predicted subtype of IRF predicted targets (a), IRF9 (b), STAT1 (c), HNF1B regulon (d), HNF1B probes (e) and HNF1A probes (f). (TIFF 1117 kb) [file 12885_2016_2540_MOESM5_ESM.tiff]
